# Shape oscillations of single blood drops: applications to human blood and sickle cell disease

**DOI:** 10.1038/s41598-018-34600-7

**Published:** 2018-11-14

**Authors:** Vahideh Ansari Hosseinzadeh, Carlo Brugnara, R. Glynn Holt

**Affiliations:** 1 Department of Mechanical Engineering, Boston University, 110 Cummington Mall, Boston, MA 02215 USA; 2 Department of Laboratory Medicine, Boston Children’s Hospital, Harvard Medical School, 300 Longwood Avenue, Boston, MA 02115 USA

## Abstract

Sickle cell disease (SCD) is an inherited blood disorder associated with severe anemia, vessel occlusion, poor oxygen transport and organ failure. The presence of stiff and often sickle-shaped red blood cells is the hallmark of SCD and is believed to contribute to impaired blood rheology and organ damage. Most existing measurement techniques of blood and red blood cell physical properties require sample contact and/or large sample volume, which is problematic for pediatric patients. Acoustic levitation allows rheological measurements in a single drop of blood, simultaneously eliminating the need for both contact containment and manipulation of samples. The technique shows that the shape oscillation of blood drops is able to assess blood viscosity in normal and SCD blood and demonstrates an abnormally increased viscosity in SCD when compared with normal controls. Furthermore, the technique is sensitive enough to detect viscosity changes induced by hydroxyurea treatment, and their dependence on the total fetal hemoglobin content of the sample. Thus this technique may hold promise as a monitoring tool for assessing changes in blood rheology in sickle cell and other hematological diseases.

## Introduction

Sickle cell disease is one of the most commonly inherited diseases worldwide with over 250,000 new births each year, and some 70,000–100,000 affected patients in North America^1^. In SCD, a point mutation occurs in the gene responsible for the production of the β-chain in hemoglobin (Hb), the main protein in red blood cells (RBCs) that is responsible for oxygen transport^2^. This mutation changes the hydrophilic glutamic acid to a hydrophobic valine amino acid residue in the β-globin chain gene, giving rise to hemoglobin S (HbS), a variant form of Hb. Under certain physiological conditions (e.g., deoxygenation) the sickle hemoglobin (HbS) can polymerize into long strands, resulting in stiffened and often sickle-shaped red blood cells^3^. Exposing cells to normal oxygen environment (normoxic) melts the fibers, but permanent and irreversible damage results from repeated exposure to alternating low-oxygen and normoxic environments^4–7^ and this may be a consequence of abnormal entry of Ca into the sickle red blood cell^8–11^. This irreversible membrane damage leads to stiffer sickle RBCs, which are unable to unsickle after re-exposure to room air (irreversibly sickled cells)^12,13^. These RBCs may adversely affect blood flow^14^ and contribute to vessel occlusion, poor oxygen transport, and hemolysis^15,16^. Vasoocclusion is the major cause of morbidity and mortality in SCD, followed by ischemia and/or ischemia/reperfusion cycles in various tissues, leading ultimately to progressive end organ damage^17,18^. Hydroxyurea (HU) has been until very recently the only FDA-approved drug for treating SCD^19^. Studies show that HU-treatment increases fetal hemoglobin (HbF) production in RBCs^20–23^ which inhibits the polymerization of HbS. However, several other additional mechanism of actions have been postulated for HU, including changes in endothelial NO metabolism.

Various techniques have been used to study the rheological properties of SCD blood and to monitor HU treatment effectiveness on SCD care, such as cone-plate rheometry^22^, co-axial cylinder viscometer^24^, tube viscometry^18^, common-path interferometric microscopy^19^, Raman microscopy^25^ and microfluidics flow resistance^26^. All these methods involve direct contact with the sample: thus, the measurements may be affected by the contact. For example, the clotting cascade can be artificially initiated^27^; local adhesion and resultant deformation of cells at the contact region can alter the cells functionally^28^.

A classical solution to the contact problem has been to employ field-based levitation as a container-less method to position and manipulate cells^29^ and biological samples^30^. Besides elimination of the contact effect, the levitation method also prevents any chemical and thermal contamination, i.e., adsorption from contact between sample and external objects^31^. The use of levitated sample droplets also has the added advantage of increased detection sensitivity since no walls disturb detection^32^. Optical interference at the walls of the container, which hampers detection when spectroscopic techniques are used, can be avoided using levitation^32^. Furthermore, compared to the chip approach^33^ to miniaturization, the levitation technique exhibits similar benefits of diversity of application and low reagent and sample consumption, while also eliminating the risk of analyte adherence to walls and interfaces^32,34,35^.

Subramanian *et al*. used magnetic levitation to measure metal-amplified changes in the density of beads labeled with biomolecules^36^. Andersen *et al*. detected cell-membrane-bound and soluble antigens in magnetically levitated cells^37^. Engineered as a compact and portable unit, magnetic levitation was used to distinguish sickle RBCs from normal cells^38^. Jung *et al*.^39^ optically characterized sickle cells using quantitative phase imaging. Zhong *et al*.^40^ used optical tweezers to trap and manipulate red blood cells *in vivo*. Acoustic levitation has long been employed to infer sample material properties^41^. Holt *et al*.^42^ utilized acoustic levitation to study the hemostasis of drops of whole blood. Using acoustic levitation coupled with Raman spectroscopy, Puskar *et al*. studied malaria-infected cells^29^. Omrane *et al*. used the third and the fourth harmonic of a Nd:YAG laser to investigate elastic, fluorescence and phosphorescence signals from the levitated droplet and measure diameter variation (surface area), mixture concentration and temperature of the drop^31^. Lopez-Pastor *et al*. also levitated drops to monitor reactions in ionic liquids^43^.

In this paper, we use acoustic levitation to study normoxic (normal level of oxygen) rheological properties of normal and SCD blood. A drop of whole blood is levitated in an acoustic field and excited to modal shape oscillation by amplitude modulation of the field. Using a laser scattering method, the free decay of the shape oscillation is measured, from which blood viscosity is inferred. Correlation of the measured viscosity with clinically measured parameters for SCD patients indicates that this technique can provide insights into the disease pathophysiology and the effectiveness of HU treatment. In particular, our results show that HU treatment reduces whole blood viscosity, and further that this viscosity reduction correlates with HbF production while exhibiting no correlation with mean corpuscular volume (MCV).

## Results

### Normal blood assay as a validation of technique

**Figure 1:**
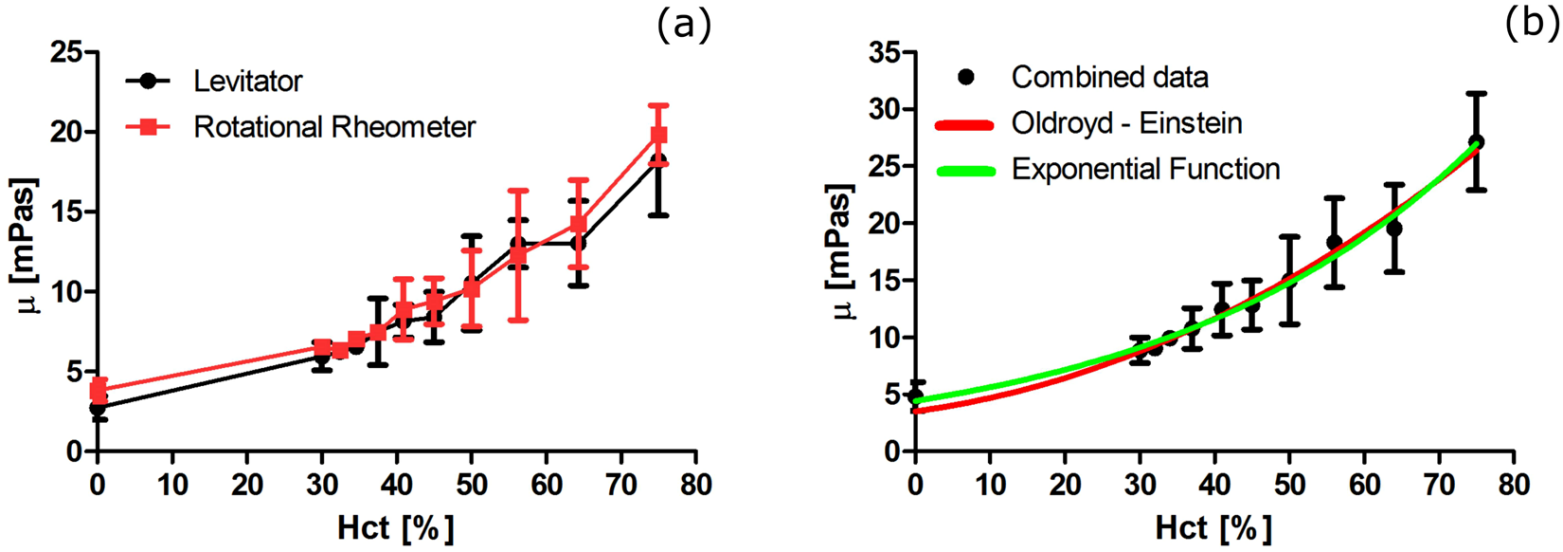
(**a**) The inferred viscosity of normal blood from a single volunteer by levitation (black) and rotational rheometer (red) measured at 37 °C and corrected to 23 °C to facilitate comparison with later results. Each point shows the average of 8 different drops (5–10 ml each) for the levitator, and 3 different samples (270 ml each) for the rheometer. Error bars in both cases indicate one sample standard deviation. The lines connecting the data are only guides for the eye. (**b**) Comparison of combined levitator and rheometer data (solid symbols), and the Oldroyd-Einstein Equation (1) with µ_0_ = 3.5 mPas, and β = 28.8 mPas.

Acoustically levitated drops are excited to shape oscillation by amplitude modulation of the acoustic pressure. When the modulation is turned off, the drop undergoes free-decay oscillations. The free-decay technique has been calibrated in our previous study for viscous liquids^44^. In this study we designed an assay that quantified the viscosity of whole blood versus hematocrit (Hct), the main factor affecting blood viscosity^45^. We performed the assay with only one volunteer’s blood (AA blood), to eliminate the existent variation between different biological samples. To validate our technique, blood viscosity was simultaneously and independently measured by cone-plate hybrid rheometer. Figure 1(a) shows the measured effective viscosity of normal AA blood by levitation and rotational rheometer. The results are in good agreement for all ranges of Hct.

The relationship between blood viscosity and Hct has been previously empirically described by an exponential function as A*exp*(k_1_Hct − k_2_T) in which A, k_1_, and k_2_ are constants. Hct and T are hematocrit and temperature °C, respectively^46,47^. Along with this empirical formula, we use the Oldroyd-Einstein^48^ formula (Equation (1)), which describes the effective viscosity of a dispersion of rigid spheres of concentration *Ф* in a Newtonian host fluid. In this way, the fit parameters can be directly used as the sample properties. In the theory, µ_0_ and *Ф* are the suspending medium viscosity and the dispersed-phase (RBC) volume fraction, respectively. For blood, µ_0_ and *Ф* represent the plasma viscosity and Hct, whereas β expresses the rate of viscosity variation vs. Hct.

On the assumption that both rotational rheometer and levitation techniques yield (to within experimental uncertainty) essentially the same result, we pooled all normal blood data and fit them with the empirical and Oldroyd-Einstein formule, and the results are shown in Fig. 1(b). Equation (1) accurately fits the measured relationship between viscosity and Hct with the fit parameters µ_0_ = 3.5 ± 0.6 mPas, and β = 28.8 ± 5.0 mPas for T = 23 °C.

1 $$\mu ={\mu }_{0}(1+\frac{5}{2}\varphi )+\beta {\varphi }^{2}$$

### Application of levitation technique to SCD blood

**Figure 2:**
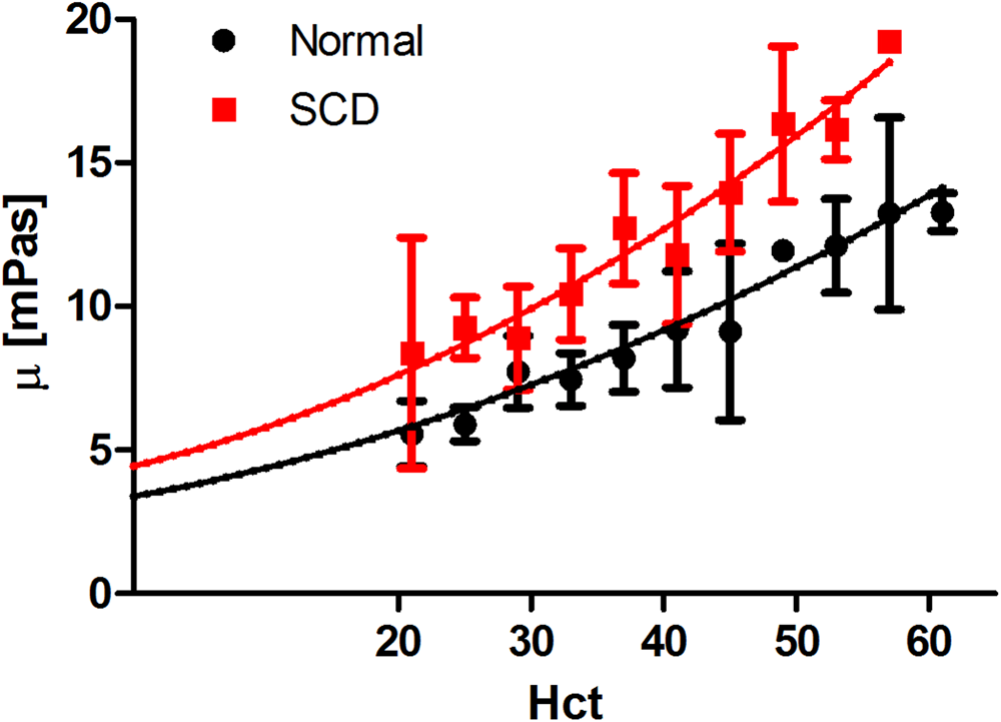
The measured viscosity at room temperature (23 °C) of 45 normal and 22 SCD blood samples using the levitator technique. Error bars show standard deviation, which shows variation among each sample. The curves show Oldroyd-Einstein fit for each data set to obtain characteristic values of µ_0_ and β. µ_0_ = 3.4 ± 0.6 mPas and β = 15.0 ± 5.6 mPas are parameters of the fitted curve of normal samples, µ_0_ = 4.4 ± 0.9 mPas and β = 23.8 ± 9.1 mPas are parameters of the fitted curve of SCD samples.

Now that levitator results have been validated by rotational rheometer, the effective viscosity of normal and SCD blood were measured and compared over a wide range of Hct values (Fig. 2). We varied Hct in each of the samples by manipulating the ratio of RBCs to plasma and obtained 3 different Hct values for each sample (more details in “Methods” section). Our experiments on 45 normal and 22 SCD samples show that at each Hct, SCD blood showed increased viscosity compared with normal blood. The fit parameters are as follows: µ_0_ = 3.4 ± 0.6 mPas and β = 15.01 ± 5.6 (normal samples), µ_0_ = 4.44 ± 0.9 mPas and β = 23.8 ± 9.1 (SCD samples).

### Effects of HU treatment on rheological properties

**Figure 3:**
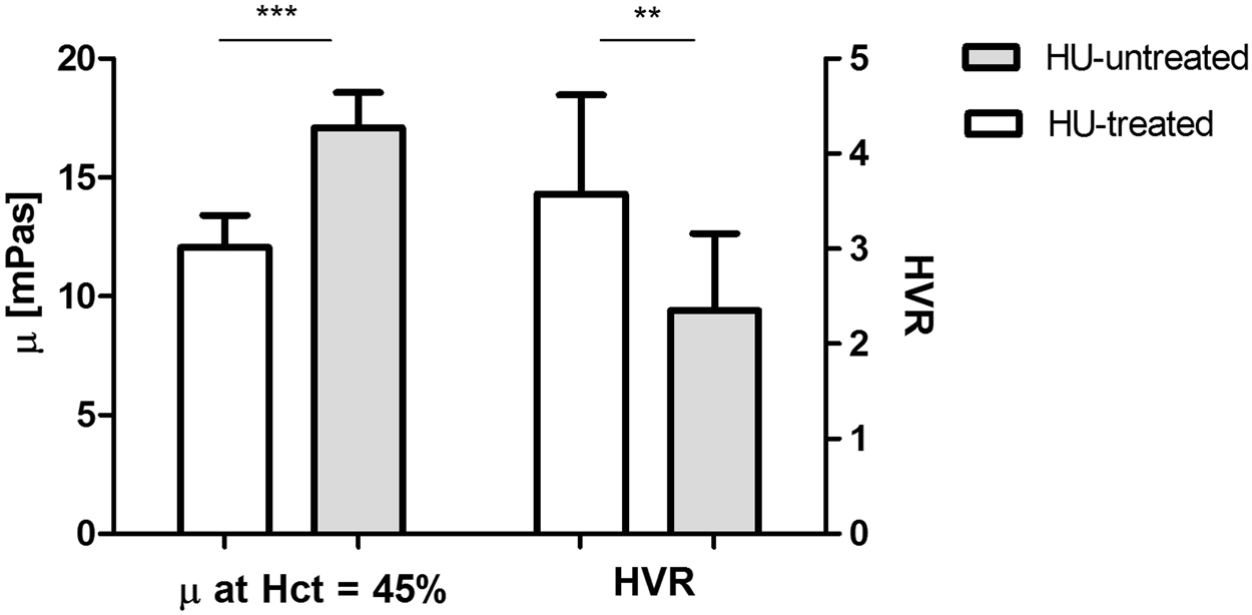
Adjusted (Equation 1) blood viscosity at Hct = 45% and HVR for both “HU-treated” and “HU-untreated” patients. Error bars show standard deviation. Standard two-tailed t tests were used to determine the significance of the difference between two groups of data, where P < 0.001 and P < 0.01.

To assess the effect of HU on blood properties, we compared the properties from patients “HU-treated” and “HU-untreated”. We used only samples from patients on stable HU treatment, meaning that they had reached therapeutic dose and were not exposed to dose changes. We did not use HU *in vitro* in any of the experiments. We varied Hct in each of the samples by manipulating the ratio of RBCs to plasma. By determining viscosity at 3 different Hct values for each sample, then fitting each sample with the Oldroyd-Einstein model Equation (1), we were able to extrapolate viscosity at a desired Hct value. Because of the strong influence of the erythrocyte concentration on the viscosity of blood, blood viscosity values are often normalized to a standard Hct of 45% by a regression equation^49^, using 3 separate Hct values for each sample. In this study we used Equation (1) to calculate viscosity at 45%. As shown in Fig. 3 HU treatment results in a significantly lower blood viscosity and this is consistent with previous studies^21,50,51^.

Studies show that the oxygen transport efficiency of SCD blood defined by Hct-to-viscosity ratio (HVR)^6,52^ may be a more reliable indicator in assessing the response to HU treatment^53^. Because the oxygen-carrying capacity of blood is directly related to Hct value, and resistance to blood flow is inversely proportional to viscosity, HVR has been used as an estimate of “oxygen transport index”^6^. Figure 3 shows significant increase of HVR for “HU-treated”- compared to “untreated” patients, consistent with earlier results^22^; this increase mirrors the corresponding decrease of the β parameter (Equation (1)) with HU treatment. Normal blood would exhibit an HVR of about 5 [1/mPas] based on Fig. 2 data.

### Correlation of rheological properties with clinical measurements for HU treatment

**Figure 4:**
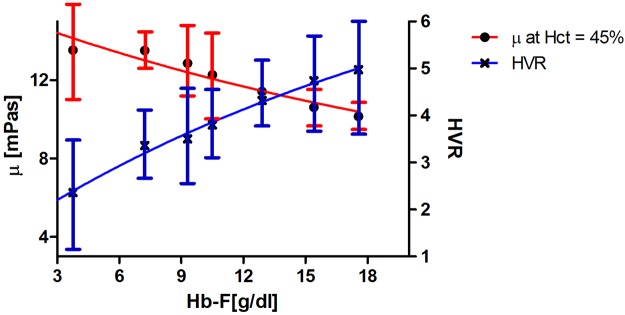
The adjusted viscosity at Hct = 45% (filled circles) and HVR (crosses) for SCD blood. Polynomial fits to the data are shown for both viscosity (Red) and HVR (Blue) as a guide for the eye. Same samples as Fig. 3. By increasing HbF, the adjusted viscosity decreases (P < 0.01). The inferred Hct to viscosity ratio or oxygen delivery index versus HbF shows HVR increases by HbF (P < 0.0001).

Fetal hemoglobin induction^20–22^ and increased MCV^19,21,22^ have been reported as two major effects of HU treatment, but there are limited reports of the rheological effects of these results. Figure 4 shows that there is a correlation between HbF and blood viscosity. Patients with higher HbF (expressed as total concentration of Hb F in g/dl of blood) have lower adjusted viscosity, and thus a higher oxygen transport index HVR. This result is in agreement with previous studies, which reported the anti-sickling effect of HbF on individual RBC^18,54^, which increases RBC deformability^55^. HU treatment has been also shown to decrease haemolysis and free hemoglobin in plasma^7,56–60^.

**Figure 5:**
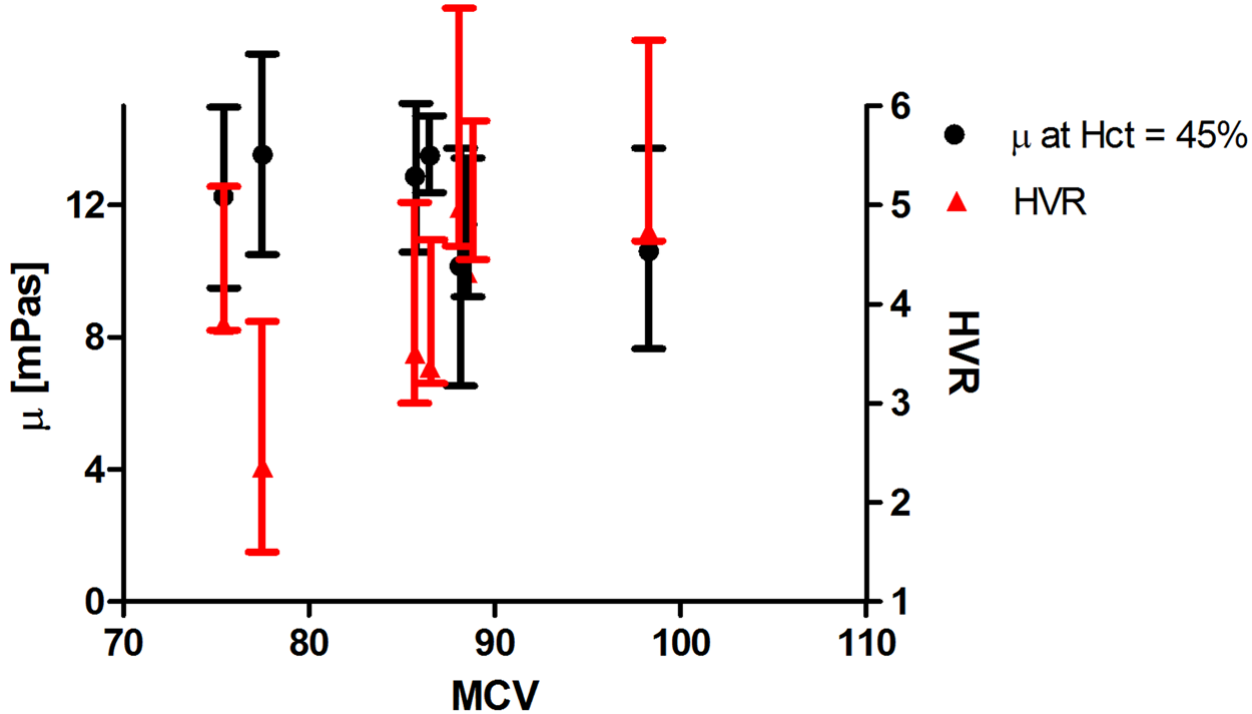
The adjusted viscosity at Hct = 45%, and HVR for SCD blood vs. MCV. No significant correlation was observed (P > 0.05).

On the other hand, we did not observe any significant correlation between MCV and our measured rheological properties illustrated in Fig. 5. Hct, and the corresponding variable *Ф* in Equation (1), are understood to be volume concentrations. As such, Hct = *Ф* ~ m × MCV, where m is the number density of RBCs in a given sample volume. Thus, to compare samples with varying MCV as in Fig. 5 for a fixed Hct, m would have to be varied controllably. But this is the implicit assumption of the Oldroyd-Einstein model, where concentration *Ф* is all that matters to the viscosity. Thus the apparent lack of correlation of viscosity with MCV in Fig. 5 seems to support the application of the rigid sphere Oldroyd-Einstein model.

## Discussion

Based on the comparison with a commercial rheometer, we have shown in this study that acoustic levitation is capable of measuring the viscosity of blood over a wide range of Hct. Our results, especially Fig. 2, indicate that the acoustic levitation-based measurement of whole blood viscosity may serve as a convenient biomarker for the altered rheology of SCD. This marker is rapidly attainable from a single drop of blood. Additionally, we demonstrate that drop shape oscillations can measure changes in viscosity that are associated with the clinical use of hydroxyurea. We also show that these changes in viscosity are correlated with the total amount of HbF present in each sample, while they are independent of the increase in MCV that is observed with hydroxyurea therapy. Thus, this technique may hold promise for assessing changes in blood viscosity in sickle cell and other hematological diseases.

There is a growing recognition that a variety of pathological conditions can lead to changes in blood viscosity. Medication-induced states^61^ and bacterial or viral infections^62^ have been shown to change viscosity. Although various techniques can be used for the measurement of rheological properties of biological fluids, acoustic levitation is unique in being capable of providing rheological information for biological samples in a minimally invasive way only by using one single drop of the sample.

The results shown in Fig. 2, taken together with those of Figs 3, 4 and 5 indicate that the increased viscosity of whole blood in SCD is not simply due to changes in RBC volume and cellular dehydration. Membrane damage is most likely an important determinant of the individual RBC membrane viscosities which must play a role in the overall blood viscosity. We are intrigued that plasma viscosity (represented by µ_0_ in Equation (1)) may also contribute, at least in theory, to determine SCD blood viscosity. Future studies will address the role of these variables. The dependence of viscosity on HbF shown in Fig. 4 and the overall improved viscosity of samples collected from patients on stable HU therapy provide a strong indication that our experimental approach reflects the pathophysiology of the disease and could be used to quantify beneficial changes induced by HU or other therapies.

## Methods

### Principles of acoustic levitation and laser scattering technique

Our experiments are performed with a single-axis acoustic levitator, a custom-built half-wave aluminum stack resonance actuated by piezoceramic (PZT) transducers, with a nominal frequency of 29 KHz. By adjusting the stack to its resonance, a standing wave is formed in the air gap between the stack and a flat reflector placed a full wavelength away. The levitator is placed inside a housing which provides temperature and humidity control. All blood assays have been performed at relative humidity of 90 ± 10. 0.9%. Sodium Chloride Isotonic Saline is used as the aerosolized humidification material, to prevent osmolality changes in the levitated blood drop.

**Figure 6:**
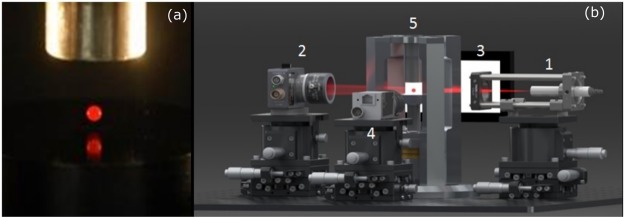
(**a**) A 2 mm diameter (long axis) drop of whole blood is levitated in the resonant standing acoustic field in the air gap between the radiator (black bottom) of the Langevin stack and the reflector (bare Al cylinder top). (**b**) Schematic of the entire experiment. A drop of whole blood is levitated in the acoustic field (5). An expanded He-Ne laser beam (1) is incident on the drop, and a photodetector (2) senses the resultant fluctuating light intensity. The backlit drop by a light source (3) is monitored by a digital camera (4).

A single 5–10 µl drop is deployed manually near one of the pressure nodes via syringe and needle. An expanded He-Ne laser beam is incident on the drop, and a photodetector situated directly in the forward scattering senses the resultant fluctuating light intensity. To decrease sensitivity to ambient light, a narrow 632.8 nm optical bandpass filter is placed in front of the photodiode. To further increase our signal to noise ratio, a vertical slit of width 300 microns is placed in front of the optical bandpass filter. Figure 6 shows a blood drop levitated in the acoustic field and a schematic of the experiment setup.

**Figure 7:**
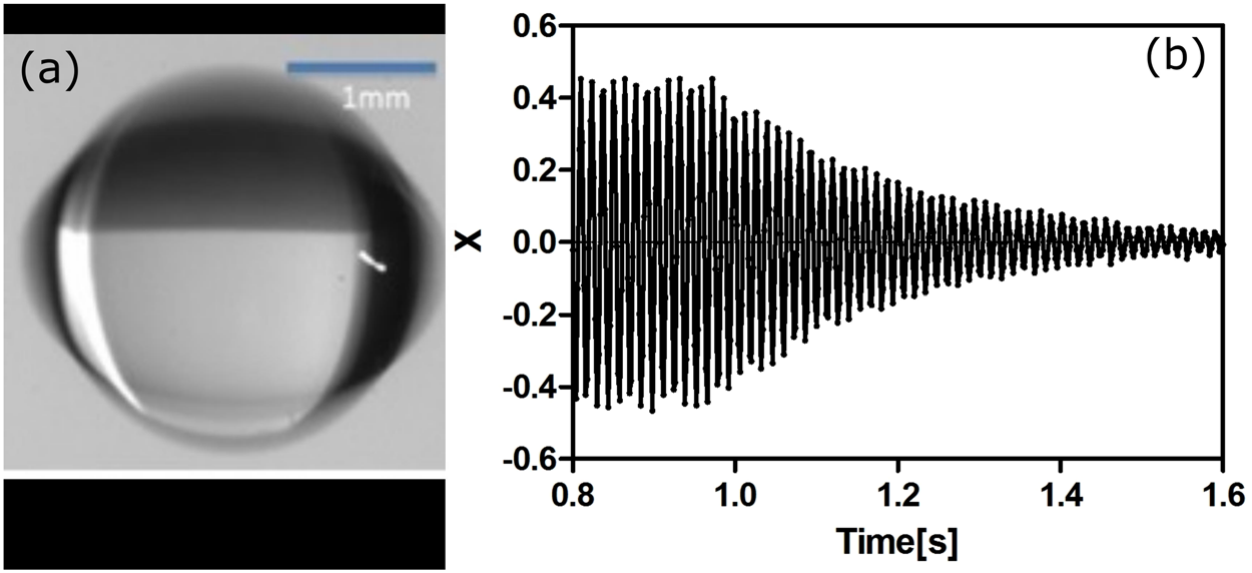
(**a**) Time-average (>1 ms exposure time) image of a 1.4 mm radius drop of 20% glycerol in water solution undergoing large-amplitude (for illustration), resonant quadrupole modal shape oscillations with f = 75 Hz. (**b**) Normalized photodetector voltage vs time.

Shape oscillations of the levitated drop are excited by amplitude-modulating the transducer drive signal at the frequency of the axisymmetric n = 2 ‘quadrupole’ mode of the drop, on the order of 100 Hz. The output voltage of the photodiode is linearly proportional to the fluctuating area of the drop shape^63^. The backlit drop is monitored during the oscillation by a digital camera. In order to maintain very small oscillations (less than 0.5% oscillatory strain amplitude) we do not use the camera images for dynamic shape oscillation data. More detailed descriptions of this technique can be found in an earlier publication^44^. Figure 7(a) shows a levitated drop undergoing very large amplitude quadrupole mode shape oscillations for illustration. Figure 7(b) shows the raw photodetector voltage data from a freely decaying shape oscillation obtained by first maintaining steady-state resonant modal shape oscillations (0.8–1 s), then allowing the oscillations to decay by turning off the modulation (1–1.6 s).

### Mechanical models

In order to infer material properties, we first apply the single degree of freedom harmonic oscillator model to the shape oscillations of drops in our experiments. Since the harmonic oscillator is a linear 2nd order ODE, we can obtain two generic oscillator parameters, resonance frequency ω_n_ and damping ratio ζ. “n” shows the mode number of oscillation. In the free decay (FD) method a drop is initially excited into steady-state quadrupole (n = 2) oscillations, whereupon the modulation is turned off and the drop transient response x is recorded (Fig. 7(b)). By fitting the experimental decay curve to the analytical decay in Equation (2) we may obtain best-fit estimates of ω_n_ and ζ.

2 $$x(t)=A{e}^{-\zeta {\omega }_{n}t}\,\cos ({\omega }_{n}\sqrt{1-{\zeta }^{2}}t)$$

Knowing the resonance frequency ω_n_, damping ratio ζ and weighing the drop after each experiment as an alternative to using digital imaging for determining R, viscosity is obtained from Equation (3)^64^. R is the drop mass-equivalent radius. Blood density ρ as a function of Hct can be found from^65^.

3 $${\mu }=\frac{{\zeta }\rho {{\rm{R}}}^{2}{{\rm{\omega }}}_{2}}{5}$$

### Blood samples preparation

Left-over material from blood samples collected for hematology studies was used for these experiments under a Human Study Protocol approved by the Institutional Review Board of Boston Children’s Hospital with a waiver of consent. The samples were anticoagulated with ethylenediaminetetraacetic acid (EDTA, 1.5 mg/ml), stored at 4 °C after the clinically required tests were performed, and used for these studies within 2 days of collection. All experiments were performed at room air and thus did not impose any additional sickling on the one present in the sample when collected. The native Hct was measured by a haematology analyzer. Hct of the samples was varied by centrifuging the samples for 5 min at 6 × 1000 min^−1^ speed, and then removing or adding autologous plasma. For example, for the experiments presented in Fig. 2, the blood sample from each subject was divided into 3 aliquots. One aliquot with native Hct was not manipulated and run as such. Two other aliquots were gently spun and the supernatant plasma was subtracted in appropriate amounts from one aliquot and added to the other one which produced samples with increased or decreased Hct, respectively. Red cells were gently resuspended and Hct was measured in each tube before being tested in the levitator.

### Rheometer measurements

A cone-plate rheometer coupled to a refrigerated recirculating fluid bath to control the temperature of the sample was used for rheological measurements. The rheometer equipped with a CP-20 cone (HR-2, TA instrument, MA) measured viscosity of whole blood within the shear rate of 0.01–1000 1/s.
